# Acute Life Interventions, Goals, and Needs Program: Social Determinants of Health Among the Most Vulnerable

**DOI:** 10.1093/geroni/igaa057.1267

**Published:** 2020-12-16

**Authors:** Lizette Munoz, Blair MacKenzie, Audrey Chun, Shamsi Fani, Susana Lavayen, Stephanie Chow

**Affiliations:** 1 Mount Sinai Hospital, New York, New York, United States; 2 Icahn School of Medicine at Mount Sinai, New york, New York, United States; 3 Mount Sinai Medical Center, New York, New York, United States; 4 Icahn School of Medicine at Mount Sinai, New York, New York, United States

## Abstract

The Acute Life interventions Goals and Needs program(ALIGN) at Mount Sinai Hospital in New York City, is an inter-professional team dedicated to offering temporary intensive ambulatory care services to the most complex older patient population. This allows us to care for the most vulnerable population which often incur multiple hospitalizations, emergency room visits. Mr.C is a 81 yo male with past medical history of Chronic COPD, Depression, Gait instability, Mild Neuro-cognitive disorder, Hearing Loss, Coronary artery disease. Most significantly he had 3 ED visits, 1 admission, where he was found on the floor of his apartment after two days, by a meals on wheels volunteer. Team conducted a comprehensive assessment of Mr.C’s social determinants of health and compiled a care plan. We learned that Mr.C does not like to bother others therefore found it difficult to seek help. Team built intensive rapport and gained his trust to help simplify medications, increase engagement and explore barriers to home care. Mr.C was connected to several community agencies including, meals on wheels for more stable food access, psychiatry to discuss depression and isolation, adult protective services for deep cleaning,financial management, pharmacy for blister packing, home care services and case management to continue encouragement with care plan. Mr.C is now able to reach out to the team as needed and has a navigator to help with managing care. This is one of many cases ALIGN encounters, that often go undetected due to comprehensive inter-professional care needed and minimal time given in traditional primary care.

